# Glycosides as Potential Medicinal Components for Ulcerative Colitis: A Review

**DOI:** 10.3390/molecules28135210

**Published:** 2023-07-04

**Authors:** Yating Niu, Jun Zhang, Dianhua Shi, Weibiao Zang, Jianguo Niu

**Affiliations:** 1 School of Basic Medical Science, Ningxia Medical University, Yinchuan 750004, China; 2 Shandong Academy of Chinese Medicine, Jinan 250014, China; 3 School of Pharmacy, Ningxia Medical University, Yinchuan 750004, China

**Keywords:** ulcerative colitis, epidemiology, glycosides, inflammatory response, oxidative stress, immune response, signal transduction

## Abstract

Ulcerative colitis (UC) is a chronic, non-specific disease of unknown etiology. The disease develops mainly in the rectum or colon, and the main clinical symptoms include abdominal pain, diarrhea, and purulent bloody stools, with a wide variation in severity. The specific causative factors and pathogenesis of the disease are not yet clear, but most scholars believe that the disease is caused by the interaction of genetic, environmental, infectious, immune, and intestinal flora factors. As for the treatment of UC, medications are commonly used in clinical practice, mainly including aminosalicylates, glucocorticoids, and immunosuppressive drugs. However, due to the many complications associated with conventional drug therapy and the tendency for UC to recur, there is an urgent need to discover new, safer, and more effective drugs. Natural compounds with biodiversity and chemical structure diversity from medicinal plants are the most reliable source for the development of new drug precursors. Evidence suggests that glycosides may reduce the development and progression of UC by modulating anti-inflammatory responses, inhibiting oxidative stress, suppressing abnormal immune responses, and regulating signal transduction. In this manuscript, we provide a review of the epidemiology of UC and the available drugs for disease prevention and treatment. In addition, we demonstrate the protective or therapeutic role of glycosides in UC and describe the possible mechanisms of action to provide a theoretical basis for preclinical studies in drug development.

## 1. Introduction

Inflammatory bowel disease is a chronic inflammatory disease of unknown etiology, including UC and Crohn’s disease [1]. UC mainly involves the rectum and sigmoid colon, occurring mostly in the mucosal and submucosal layers of the colon and less frequently in the muscular layer, in continuous distribution and with slowly recurrent episodes [2]. The main clinical manifestations are abdominal pain, diarrhea, and purulent stools [3]. Some patients with UC may also have extra-intestinal involvement of joints, spine, bile ducts, skin, eyes, and mouth, resulting in a range of extra-intestinal symptoms. Eventually, complications such as acute peritonitis and intestinal perforation may occur, increasing the risk of hospitalization, surgery, and cancer [4,5].

As a chronic recurrent immune disease, UC is thought to result from the dysregulated expression of molecules involved in pro- and anti-inflammatory processes, often in association with other autoimmune diseases. Although the pathogenesis of epithelial damage due to an abnormal inflammatory response is unknown, it is speculated that its etiology may be related to factors such as diet, genetics, external environment, and intestinal flora [6,7]. UC has been reported to be particularly prevalent in Western developed countries and relatively uncommon in developing countries. However, changes in the standard of living and dietary habits of people in developing countries have led to a global increase in the incidence and prevalence of UC in recent years [8]. Currently, the treatment of UC is mainly divided into surgical and non-surgical therapies. Non-surgical treatments are mostly used in clinical practice and include drugs such as aminosalicylates, immunosuppressants, glucocorticoids, and biologics. However, many of these drugs are limited in clinical application, and their clinical efficacy is unstable and may even bring about a series of problems such as hepatic and renal toxicity, drug dependence, and recurrence of the disease after withdrawal of the drugs [9,10,11]. When drug therapy is ineffective, 15% of patients still need to undergo colorectal resection when it is ineffective at the later stage. Up to now, no effective treatment options for UC have been found to compensate for the shortcomings of conventional treatment [12]. Thus, we need to look for safer and more effective drugs. The bioactive compounds derived from natural products, especially medicinal plants, are emerging as new therapies for a variety of diseases, including those affecting the gastrointestinal tract; these products have produced encouraging results and reduced adverse reactions. The multiple biological activities of plants are due to the diversity of secondary metabolites that bind to macromolecules in the organism. These are grouped according to their synthesis pathway into classes such as alkaloids, flavonoids, and terpenes, among others [13]. As for glycosides, they are ubiquitous natural products, and due to their structural diversity, they therefore possess many pharmacological activities such as anti-inflammatory, antioxidant, and analgesic effects [14,15,16]. In this manuscript, we select 21 glycosides with relatively clear mechanisms and summarize their roles in UC to provide a reference for the drug development of glycoside molecules.

## 2. Epidemiology of UC

Epidemiological data show that there are significant geographical and ethnic differences in the incidence of UC, that it occurs in people aged 20–40 years, that it causes disability, and that it has a high rate of colon cancer [1,17,18]. The incidence of UC has reached 8.8–23.1/100,000 per year in North America; 0.6–24.3/100,000 per year in Europe; 7.3–17.4/100,000 per year in Oceania; and 2.03–17.8/100,000 per year in Asia [17]. Although the incidence of UC is much lower in developing countries such as Asia than in Western countries, the rate of increase in the incidence of UC is much higher than in Western countries [19], which is a relative reflection of the fact that the development of the disease is closely related to the social transformation of the country, the standard of living of the people, their ethnicity, and their dietary habits.

Surveys estimate that the direct and indirect costs associated with UC range from EUR 12.5 billion to EUR 29.1 billion in Europe and from USD 8.1 billion to USD 14.9 billion in the U.S. each year [20,21]. The root cause is also the fact that the exact pathogenesis of UC is not fully understood, and most of the available therapeutic drugs can only be used to alleviate the symptoms and are hardly curative. This not only adds to the national health care burden but also has a serious impact on the quality of life of patients and their families. In the 21st century, the prevalence of UC has also increased dramatically worldwide due to the proliferation of an aging population and the availability of early UC diagnosis [20]. Today, UC has been included as a global disease and has become a new world burden of disease.

## 3. Pharmacological Treatment of UC

The majority of patients are currently treated with medications, the choice of which is guided by the severity and progression of the disease [1]. In the course of UC development, drugs with low toxicity are generally chosen first, and if these do not provide the desired relief, other drugs with better results are chosen. The drugs shown in Table 1 have shown good results in clinical use for the treatment of UC, but these conventional drugs are mainly for single-target therapy, have limitations, and are associated with serious side effects.

Aminosalicylates are used to alleviate the disease by inhibiting the production of pro-inflammatory cytokines and oxygen free radicals, blocking neutrophil chemotaxis, and mast cell activation. These drugs are well tolerated by patients, but long-term use can lead to dizziness, headaches, and gastrointestinal adverse effects as well as blood disorders and male infertility as the dose increases over time [22,23]. Glucocorticoids are used for acute and severe UC as well as for mild cases that are intolerant to aminosalicylates or are refractory to treatment. Glucocorticoids can exert anti-inflammatory and immunosuppressive effects by interacting with their corresponding receptors or other nuclear transcription factors. Glucocorticoids have powerful anti-inflammatory effects but are associated with serious side effects, including metabolic disorders, osteoporosis, systemic perverse immune responses, and delayed wound healing [24,25]. For the palliative treatment of hormone-dependent UC patients, immunosuppressive drugs are used. These drugs have high hepatotoxicity and nephrotoxicity and are therefore usually used only in an adjuvant manner [26,27]. Microbial agents are used to improve mild to moderate UC symptoms by regulating the intestinal flora [28,29]. In addition, biologics can be used as appropriate in patients with acute severe UC or severe cases when immunosuppressants are ineffective, but their use is more limited due to their high cost and possible side effects such as leukocytopenia, neutropenia, and allergy [30,31,32]. This is why the search for natural products with abundant supplies and few side effects is of great interest to scholars at home and abroad.

## 4. Synopsis of Glycosides

Biologically active substances of natural origin are an important source of new drug discovery. In recent years and with improved techniques for the isolation of active ingredients from plants, many sugar-containing active ingredients from plants have been isolated and identified [33,34].

Glycosides are compounds formed when the hemiacetal hydroxyl group of a sugar loses a portion of water or other small molecules by coupling with a ligand to condense, and they consist of both a glycosyl group and aglycone [35,36,37,38,39]. There are many different ways of linking glycosides internally. According to the type of aglycone, glycosides can be classified into phenolic glycosides (e.g., salidroside **10**), flavonoid glycosides (vitexin **6**), terpenoid glycosides (asperuloside **13**), etc. [40,41]. In addition, glycosides can be divided into primary glycosides (glycosides originally present in the plant) and secondary glycosides (hydrolysis or structural change of the primary glycosides) according to the form in which they exist in the organism.

Glycosides, the main form of sugar present in nature, have multiple pharmacological activities. For example, paeoniflorin **12**, isolated from peony, has anti-inflammatory, antipyretic, anti-spasmodic and neuroprotective, and cerebral effects [42,43,44,45,46]. The polydatin **11**, extracted from the rhizomes of *Polygonum cuspidatum*, has anti-inflammatory, oxidative-stress-reducing, and apoptosis-inhibiting effects in ulcerative colitis [47]. The multiple pharmacological activities of glycosides are related to their structure–activity relationships [48]. The removal of hydroxyl groups from glycoside ligands as well as hydroxymethylation and the elimination of phenylpropenyl or phenylethyl all decrease the pharmacological activity, while the antioxidant activity is related to the number of unsaturated bonds, the number and position of phenolic hydroxyl groups, and the length of carbon chains [49]. In addition, glycosides have the characteristics of having multiple targets and mechanisms. For example, polydatin 11 regulates the HO-1/NQO1 signaling pathway through the AKT and Nrf2 pathways [50]. Paeoniflorin **12** inhibits ulcerative-colitis-related disease by targeting EGFL7 and has a protective effect on the TLR4/NF-κB signaling pathway [51].

Furthermore, the concept of glycoside drugs has been expanded from the general reference to glycoconjugate molecular drugs only to glycoside-based drugs. For example, digoxin is a representative glycosidic drug with cardiotonic effects [52]. In addition, since glycosides act mainly on the cell surface rather than inside the nucleus, they are characterized by relatively low toxic side effects and have a wide range of applications in the pharmaceutical and food fields, which are of greater importance from a medical point of view [53]. Currently, more and more scholars are turning their attention to glycoconjugates in the hope of discovering and developing new drugs with good activity for the benefit of mankind.

## 5. Anti-Inflammatory Effects of Glycosides in a Model of UC

The establishment of a suitable animal model is important for the in-depth study of the pathomechanism of UC and preclinical drug screening. There are numerous methods to study animal models of UC, mainly including chemical, immunological, transgenic, or knockout genes [54], among which chemical stimulation models are most commonly used due to their simplicity and economic feasibility. Commonly used chemical induction agents include dextran sodium sulfate (DSS), trinitrobenzene sulfonic acid (TNBS), acetic acid, oxazolone (OXZ), dinitrochlorobenzene (DNCB), etc. Different modeling agents can be selected according to the needs of the experiment [55]. Among them, DSS- and TNBS-induced UC models are the more widely used animal models due to their simple modeling method, good reproducibility, and clinical symptoms and pathological changes that are extremely similar to those of human UC [56,57].

Under normal conditions, the intestine has an intact barrier function that prevents pathogenic antigens in the intestinal lumen from invading the organism and maintains its normal functioning. After modeling, the integrity of the colonic epithelial barrier is disrupted, which can increase the permeability of the intestinal mucosa, leading to increased expression of a range of inflammatory factors and triggering an inflammatory response [58].

**Figure 1:**
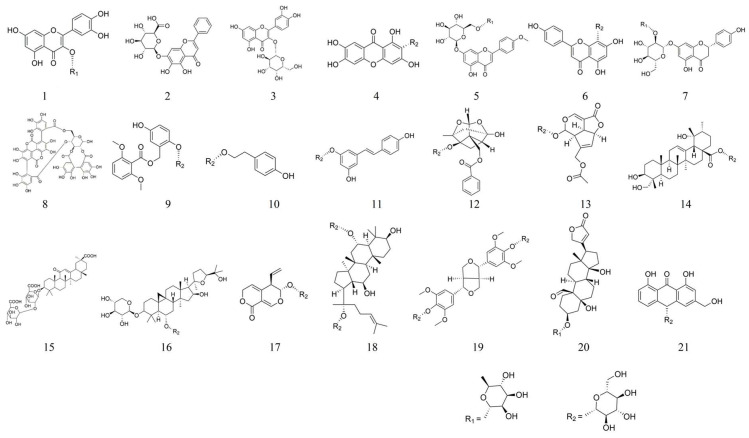
Structural formulae of several glycosides.

In this review, we searched the literature on glycosides in the last decade by using *PubMed*, *Google Scholar*, and the *Chinese National Knowledge Infrastructure* database, from which we selected 21 glycosides that were reported for their clear chemical structures, anti-inflammatory effects, or clear pharmacological mechanisms. We also classified these 21 glycosides with significant anti-inflammatory effects and summarized their roles in UC (Table 2 and Table 3, Figure 1).

### 5.1. Quercitrin ***1***

Quercitrin **1** is a bioflavonoid derived from quercitrin **1** and is widely found in a variety of medicinal or edible plants. It shows a very wide range of biological actions, including anti-tumor, antiviral, anti-thrombotic, anti-inflammatory, anti-allergic, anti-atherosclerotic, and vasodilatory effects as well as stimulating cellular immunity [96,97,98,99,100,101,102].

It has been documented that in DSS-induced colitis in mice, treatment with quercitrin **1** (1 and 5 mg/kg) improves histopathology and reduces biochemical parameters, inflammation, and bacterial translocation [59].

Another article showed that in TNBS-induced experimental colitis, quercitrin **1** (5 mg/kg) prevented early mesenteric vascular hyporesponsiveness by reducing NO overproduction by iNOS. Thus, the mesenteric vascular bed may be a new target for the prevention of UC [60].

In summary, quercitrin **1** has anti-inflammatory and protective effects on the intestinal mucosa in experimental colitis and may be used as an alternative treatment for inflammatory bowel disease. Its role in preventing bacterial translocation and preventing early mesenteric vascular hyporesponsiveness requires further studies to elucidate.

### 5.2. Baicalin ***2***

Baicalin **2** is a flavonoid glycoside extracted from *Scutellaria baicalensis* with a variety of biological activities, including anti-inflammatory, anti-bacterial, and anti-tumor activities [103,104,105,106]. Studies have demonstrated that baicalin **2** (100 mg/kg) can improve the severity of DSS-induced colitis, and the potential mechanism is closely related to the inhibition of the TLR4/NF-κB signaling pathway [61]. Baicalin **2** (100 mg/kg) also alleviated the extent of TNBS-induced colitis in mice by inhibiting the PI3K/AKT signaling pathway [62].

Baicalin **2** (30, 60, and 120 mg/kg) was able to dose-dependently improve the severity of experimental colitis by a mechanism that may be related to the inhibition of oxidative stress and regulation of a range of proteins associated with IEC apoptosis [63]. In addition, baicalein (20, 50, and 100 mg/kg) ameliorated DSS-induced colitis by activating NLRP6 inflammatory vesicles to promote mucus secretion by cupped cells through a mechanism associated with activation of the NLRP6/IL-18 pathway to promote cupped cell production in the mucosa [64].

There is also evidence that baicalin **2** (10 mL/kg) alleviates TNBS-induced colitis in mice concerning the balance between Th17 and Treg cells [65].

In summary, we found that baicalin **2** exerts powerful anti-inflammatory effects in UC treatment by mechanisms related to reducing the release of inflammatory factors, inhibiting oxidative stress and anti-apoptosis, modulating UC-related receptors, modulating immune cells, and regulating different signaling pathways. Thus, baicalin **2** may be a promising drug for UC, and its components may be important lead compounds for the development of novel UC-related chemical drugs.

### 5.3. Hyperoside ***3***

Hyperoside **3** is a flavonoid glycoside extracted from *Hypericum* spp. and *Hawthorn* spp., with a variety of pharmacological activities including anti-inflammatory, anti-bacterial, anti-tumor, antioxidant, and immunomodulatory functions [107,108].

In DSS-induced colitis in mice, hyperoside **3** (80 and 120 mg/kg) was able to exert a protective effect by inhibiting inflammation and apoptosis, the effect of which may be related to the activation of the Nrf2 signaling pathway [66].

Hyperoside **3** (3, 10, and 30 mg/kg) attenuated DSS-induced colitis in mice in association with modulation of Th17/Treg immune homeostasis and stabilization of PPARγ levels [67].

These findings expand our understanding of the role of hyperoside **3** and may provide potential therapeutic targets for UC.

### 5.4. Mangiferin ***4***

Mangiferin **4** is a flavonoid glycoside isolated mainly from the rhizome of mango, with antioxidant, anti-inflammatory, immunomodulatory, anti-bacterial, and analgesic effects [109,110,111,112]. Mangiferin **4** (10, 30, and 100 mg/kg) reduced inflammatory changes in colonic tissue in TNBS-induced colitis in rats, and this protective effect was mainly dependent on its anti-inflammatory and antioxidant properties [68]. There is also evidence that mangiferin **4** (10 and 20 mg/kg) ameliorates inflammatory colitis in TNBS-induced colitis in mice by regulating Th17/Treg cell homeostasis and inhibiting the activation of the NF-κB signaling pathway [69].

Another article clearly showed that mangiferin **4** (50 mg/kg) was able to exert a protective effect on DSS-induced acute colitis by inhibiting the NF-κB and MAPK signaling pathways [70].

The above results provide strong evidence for the use of mangiferin **4** in the treatment of human UC.

### 5.5. Linarin ***5***

Linarin **5** is a natural flavonoid compound isolated mainly from plants such as chrysanthemum and peppermint, with anti-inflammatory, antioxidant, analgesic, antipyretic, and anti-tumor as well as sedative, neuroprotective, anti-apoptotic, and anti-osteoporotic effects [113,114,115,116]. It has been shown that linarin **5** (20 and 50 mg/kg) can improve DSS-induced colitis in mice by inhibiting the inflammatory response, maintaining intestinal barrier function, and regulating intestinal flora [71].

### 5.6. Vitexin ***6***

Vitexin **6** is mainly a flavonoid glycoside isolated from Hawthorn, with biological activities such as antioxidant, anti-inflammatory, anti-tumor, anti-hypertensive, and anti-convulsant effects [117].

Studies have shown that in DSS-induced colitis mice, vitexin **6** (20 and 80 mg/kg) can combat colitis by inhibiting intestinal mucosal inflammation, maintaining intestinal barrier homeostasis, and remodeling the intestinal flora [72].

It has also been shown that vitexin **6** (40 and 80 mg/kg) not only alleviates DSS-induced colitis in mice but also protects against colitis-induced liver damage from inflammatory responses, which relies heavily on the inhibition of TLR4/NF-κB signaling pathway activation [73].

In summary, vitexin **6** was able to alleviate not only colitis but also colitis-induced liver injury, indicating multiple pharmacological activities of the drug.

### 5.7. Naringin ***7***

Naringin **7** is a flavonoid glycoside extracted from grapefruit, lime, and citrus seeds and has a variety of biological activities, including anti-inflammatory and antioxidant effects [118,119].

Naringin **7** (25, 50, and 100 mg/kg) was shown to alleviate DSS-induced colitis, and its anti-UC activity was associated with PPARγ activation. In addition, naringin **7** significantly inhibited DSS-induced NLRP3 inflammasome activation and modulated ZO-1 expression [74].

In addition, naringin **7** (20, 40, and 80 mg/kg) also alleviated TNBS-induced colitis in rats, and its anti-UC activity was associated with antioxidant and anti-inflammatory responses [75].

The above results suggest that naringin **7** may be a potentially effective drug candidate for UC and deserves further development and exploration.

### 5.8. Punicalagin ***8***

Punicalagin **8** is a polyphenolic active ingredient extracted from pomegranate, with anti-inflammatory, antioxidant, anti-apoptotic, and anti-proliferative biological activities [120,121,122,123]. It has been shown that in DNBS-induced colitis in rats, administration of punicalagin **8** (4 mg/kg) exhibited significant anti-inflammatory activity and improved inflammatory bowel disease in rats, which may be attributed to direct inhibition of the transcription factor NF-κB [76].

Punicalagin **8** has a significant anti-inflammatory effect on colitis and therefore could be a potential drug for the treatment of colitis, the mechanism of action of which needs to be further explored.

### 5.9. Curculigoside ***9***

Curculigoside **9** is a phenolic glycoside component of *Curculigo orchioides Gaertn* that has various pharmacological activities such as anti-inflammatory, antioxidant, anti-osteoporotic, and neuroprotective effects [124,125,126,127]. It has been documented that curculigoside **9** (500 and 100 mg/kg) inhibits disease activity index, tissue damage, and cell death in DSS-induced colitis mice. It was also able to significantly reverse these alterations in iron-toxicity characteristics such as iron overload, GSH depletion, ROS and MDA production, and reduced expression of SOD and GPX4 [77]. These findings suggest that curculigoside **9** prevents iron sagging in UC by inducing GPX4, suggesting it as a potential therapeutic agent for UC.

In conclusion, in addition to alleviating the symptoms of DSS-induced colitis in mice, curculigoside **9** was also able to reverse the altered iron toxicity profile, which also suggests that the drug’s effects are diverse, while its mechanism requires further study.

### 5.10. Salidroside ***10***

Salidroside **10** is a phenolic glycoside extracted from *Rhodiola rosea*, which has been proven to have a variety of pharmacological effects, including anti-aging, antioxidant, anti-cancer, anti-inflammatory, antioxidant, and neuroprotective activities [128,129,130].

It has been shown that salidroside **10** may exert a protective effect by reducing DSS-induced colonic tissue damage in mice through activation of the SIRT1/FoxOs pathway [78].

Additional data suggest that salidroside **10** protects against experimental colitis by reversing TREM1-associated macrophage pyroptosis and gut microbiota dysregulation-derived Th17/Treg imbalance, suggesting a potential role for UC [79].

In summary, salidroside **10** offers new options as a treatment for UC and merits further drug development.

### 5.11. Polydatin ***11***

Polydatin **11** is a phenolic glucoside extracted from the traditional Chinese medicine tiger cane. Many studies have shown that it has a wide range of pharmacological activities, such as anti-fibrotic, anti-tumor, anti-atherosclerotic disease, and anti-hepatitis effects as well as protection against multi-organ ischemia-reperfusion injury and dementia-related diseases [131,132,133,134,135,136,137,138,139].

There is evidence that polydatin **11** (15, 30, and 45 mg/kg) effectively reduce colonic oxidative stress and apoptosis. This effect may be mediated by the up-regulation of the Shh signaling pathway [47,80].

In addition, it has been shown that polydatin **11** inhibits intestinal inflammation and oxidative stress and maintains intestinal epithelial barrier integrity by mechanisms related to NF- κB, MAPK, and AKT/Nrf2/HO-1/NQO1 signaling pathways [50]. Interestingly, however, the authors did not indicate in their article the exact dose of polydatin **11** used in DSS-induced colitis in mice, which needs to be further mapped out.

Polydatin **11** (30 and 60 mg/kg) also alleviates DSS- and TNBS-induced colitis by directly binding to STAT3, specifically inhibiting STAT3 phosphorylation and correcting Th17/Treg homeostasis [81].

In summary, polydatin **11** can exert anti-colitis effects through a variety of mechanisms, so we have reason to believe that polydatin may be a promising candidate for the treatment of UC.

### 5.12. Paeoniflorin ***12***

Paeoniflorin **12** is a terpene glycoside isolated from *Paeonia lactiflora*, with pharmacological effects such as anti-inflammatory, antipyretic, anti-spasmodic, neuroprotective and cerebral, antidepressant, immunomodulatory, and anti-tumor effects as well as scavenging free radicals in the body [42,43,44,45,46,48,49,140].

It has been shown that continuous administration of paeoniflorin **12** (50 mg/kg) for 7 days significantly reduced the severity of DSS-induced colitis and led to a down-regulation of the associated inflammatory parameters, suggesting that its beneficial effects may be related to blocking the activation of NF-κB and MAPK pathways [82]. In addition, we found that paeoniflorin **12** (3 g/kg) had a therapeutic effect on AOM/DSS-induced colitis-associated cancer mice by a mechanism associated with inhibition of TLR4/NF-κB-mediated inflammatory responses and EGFL7 expression [51].

Paeoniflorin **12** (15, 30, and 45 mg/kg) also exerted protective effects against TNBS-induced colitis in mice by inhibiting inflammation and apoptosis through the MAPK/NF-κB pathway [83]. Paeoniflorin **12** (20 mg/kg) also exerted anti-UC activity by suppressing inflammatory responses and eosinophil infiltration [84].

In summary, paeoniflorin **12** can play a role in the treatment of colitis-associated cancers in addition to its protective role in UC, suggesting a diversity of drug actions that merits further in-depth study.

### 5.13. Asperuloside ***13***

Asperuloside **13** is a terpenoid extracted from Rubiaceae, Eucommiaceae, and other plants. Recent pharmacological studies have shown that asperuloside **13** has a variety of pharmacological activities that are anti-inflammatory, antioxidant, and immunomodulatory [141]. There is evidence that asperuloside **13** (125 and 0.5 mg/kg) may improve DSS-induced colitis in mice by alleviating inflammation and oxidative stress, activating the Nrf2/HO-1 signaling pathway, and limiting the NF-κB signaling pathway [85].

The above indicates that the drug is characterized by multiple pharmacological mechanisms and predicts a potential application of asperuloside **13** in the treatment of UC.

### 5.14. Pedunculoside ***14***

Pedunculoside **14** is a naturally occurring triterpene glycoside derived from the bark of iron holly. Previous studies have shown that pedunculoside **14** has anti-inflammatory, anti-tumor, anti-viral, cholesterol-lowering, and blood-pressure-lowering effects [142,143,144]. There is evidence that pedunculoside **14** (5, 15, and 30 mg/kg) has significant efficacy in DSS-induced UC, suppressing the expression of inflammatory mediators by inhibiting the activation of MAPK and AKT/NF-κB signaling pathways [86].

In summary, pedunculoside **14** has a good therapeutic effect on UC and may be a potential natural product for the treatment of UC.

### 5.15. Glycyrrhizin ***15***

Glycyrrhizin **15** is triterpenoid saponin derived from *Glycyrrhiza glabra* with anti-inflammatory, anti-ulcer, anti-hepatocytotoxic, anti-cancer, and anti-viral biological activities [145,146,147]. In a rat model of acetic-acid-induced UC, glycyrrhizin **15** (40 mg/kg) was able to attenuate the inflammatory response by inhibiting NF-κB, TNF-a, and ICAM-1 in the colonic mucosa [87]. Glycyrrhizin **15** (100 mg/kg) also exerted anti-inflammatory effects through the up-regulation of PPARc [88].

Furthermore, in TNBS-induced experimental colitis, glycyrrhizin **15** (100 mg/kg) was able to modulate the intestinal inflammatory response by regulating the subtle balance of T cells [89].

In conclusion, glycyrrhizin **15** can play a significant anti-inflammatory role in experimental colitis, and its mechanism is related to inhibiting the expression of inflammatory factors, alleviating oxidative stress, up-regulating PPARγ activity, and regulating the expression of immune cells. This provides strong evidence for glycyrrhizin **15** as a new potential therapeutic agent.

### 5.16. Astragaloside Ⅳ ***16***

Astragaloside Ⅳ **16** is a triterpenoid saponins isolated from *Astragalus membranaceus.* Studies have shown that astragaloside IV **16** has immunomodulatory, anti-fibrotic, anti-inflammatory, anti-radiation, anti-viral, antioxidant, anti-tumor, and cardiovascular protective effects [148,149,150]. Studies have demonstrated that astragaloside IV 16 (50 and 100 mg/kg) prevents DSS-induced acute colitis by remodeling Th17/Treg cell homeostasis and anti-oxidative stress, with the potential mechanism closely related to the inhibition of the Notch signaling pathway [90].

### 5.17. Gentiopicroside ***17***

Gentiopicroside **17** is a terpenoid glycoside isolated from gentian, with a variety of pharmacological activities, including anti-inflammatory, cholestatic, and anti-hepatotoxic effects [151,152].

Gentiopicroside **17** (50, 100, and 200 mg/kg) may exert anti-inflammatory effects on DSS-induced acute colitis by inhibiting the expression of inflammatory factors, suggesting a possible therapeutic potential in the treatment of colitis, but its exact mechanism of action needs further study [91].

### 5.18. Ginsenoside Rg1 ***18***

Ginsenoside Rg1 **18** is a terpenoid glycoside isolated from *Ginseng and Panax notoginseng,* which has a variety of pharmacological activities, including anti-inflammatory and neuroprotective effects, effects on obesity, etc. [153,154,155,156,157].

Ginsenoside Rg1 **18** (200 mg/kg) significantly improved DSS-induced colonic injury and colonic inflammation in mice, which may be related to the regulation of intestinal flora [92].

### 5.19. Liriodendrin ***19***

Liriodendrin **19** is one of the active ingredients extracted from *liriodendrin.* [158]. Liriodendrin **19** has a variety of biological functions, including anti-inflammatory, antioxidant, anti-tumor, anti-fungal, and anti-platelet coagulation and also has some anti-Alzheimer’s effects [159,160,161,162]. It has been shown that liriodendrin **19** (100 mg/kg) exerts anti-inflammatory activity in DSS-induced colitis in mice by inhibiting oxidative stress and activation of Akt and NF-κB pathways [93].

### 5.20. Convallatoxin ***20***

Convallatoxin **20** is a steroidal glycoside isolated from *Calendula officinalis* with a variety of pharmacological activities, including anti-inflammatory, antioxidant, anti-bacterial, anti-tumor, anti-angiogenic, and cardiotonic [163,164,165,166,167].

The ability of convallatoxin **20** (50 and 150 μg/kg) to ameliorate DSS-induced inflammation in colitis by activating PPARγ and inhibiting NF-κB suggests that it may be a promising compound for the treatment of UC [94].

### 5.21. Aloin A ***21***

Aloin A **21** is an anthraquinone glycoside extracted from the secretion of *Aloe vera* leaves and has anti-inflammatory, anti-bacterial, antioxidant, anti-viral, and anti-cancer pharmacological effects [168]. Aloin A **21** (25 and 50 mg/kg) can prevent DSS-induced colitis by enhancing intestinal barrier function through inhibition of the Notch signaling pathway [95].

## 6. Anti-Inflammatory Mechanisms of Glycosides in UC

### 6.1. Suppressing Inflammatory Responses

Excessive production of inflammatory factors and mediators such as TNF-α, IL-1, IL-6, COX-2, and iNOS in the intestine can dominate and perpetuate the inflammatory response [91]. In addition, ICAM-1 acts as a glycoprotein that mediates cell–cell and cell–extracellular matrix adhesion and is proportional to the severity of the inflammatory response when UC occurs [169].

All the glycosides in Table 3 exerted anti-inflammatory effects by inhibiting the expression of inflammatory factors and mediators in the colonic tissues of UC mice to varying degrees. Therefore, the study of the effects of drugs on inflammation-related factors and mediators is important for the treatment of UC.

### 6.2. Reduction of Oxidative Stress

Oxidative stress is thought to be one of the etiologies involved in inflammatory bowel disease. Excessive oxidative reactions can upset the balance of redox reactions in the colonic mucosa and cause intestinal damage. Whereas antioxidant enzymes are markers for scavenging free radicals generated by oxidative stress, drugs can alleviate the intestinal oxidative stress state in UC by increasing their expression levels [170].

**Figure 2:**
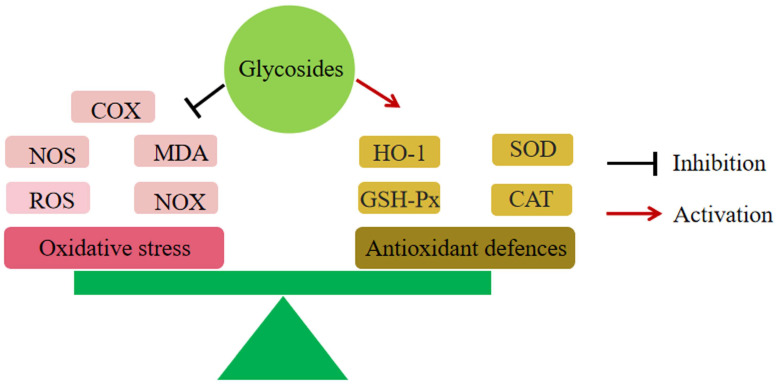
Glycosides and oxidative stress. All glycosides produced relief of UC by inhibited oxidative stress.

All the glycosides in Table 3 were able to reduce intestinal pathological damage and improve clinical symptoms of UC by modulating the expression of different antioxidant enzymes (Figure 2).

### 6.3. Anti-Apoptosis

The Bcl-2 protein family consists of Bcl-2, Bcl-xL, and Bax. In animal models, overexpression of Bcl-2 attenuates joint damage in animals, while overproduction of Bax promotes apoptosis.

In addition, Bcl-2 blocks the release of cytochrome c and down-regulates caspase activity. Caspase-3 is a central molecule in apoptosis, and its activation is regulated by a series of signal transduction cascades. Moreover, caspase-9 can be activated through the Bcl-2/Bax-ratio-mediated apoptotic pathway [83].

**Figure 3:**
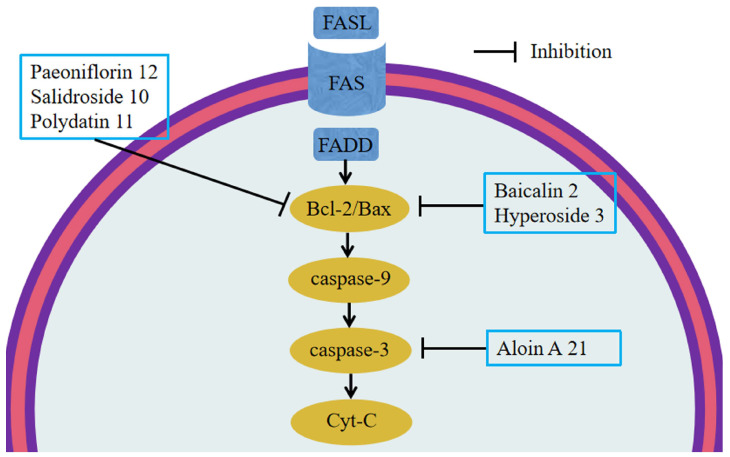
Glycosides and apoptosis. Salidroside **10** and polydatin **11** down-regulated the expression of Bax, caspase-3, and cleaved caspase-3 and up-regulated the expression of Bcl-2. Paeoniflorin **12**, baicalin **2**, and hyperoside **3** can down-regulate Bax. Aloin A **21** down-regulated cleaved caspase-3 expression.

Studies have shown that salidroside **10** and polydatin **11** inhibit apoptosis in colon cells by down-regulating the expression of Bax, caspase-3, and cleaved-caspase-3 and up-regulating the expression of Bcl-2 [47,78]. Paeoniflorin **12**, baicalin **2**, and hyperoside **3** can also inhibit colon cell apoptosis by down-regulating Bax expression. Aloin A **21** also inhibited apoptosis in colon cells by down-regulating cleaved-caspase-3 expression [95]. This suggests that apoptosis plays a role in the pathogenesis of UC [63,66,83] (Figure 3).

### 6.4. Regulation of Impaired Intestinal Epithelial Barrier Function

The mucus proteins secreted by the cupulae cover the intestinal epithelium to form a dense mucin network that forms the first barrier in the intestinal lumen [171]. In addition, the tight junction (TJ) is the most important component of the intestinal epithelial barrier and is a complex of claudins and occludin proteins, peripheral membrane protein family ZOs, and other proteins. Abnormal expression of TJ can increase the permeability of the intestinal epithelial barrier, leading to the entry of pathogenic antigens such as bacteria into the mucosa and blood circulation, causing inflammation [172].

**Figure 4:**
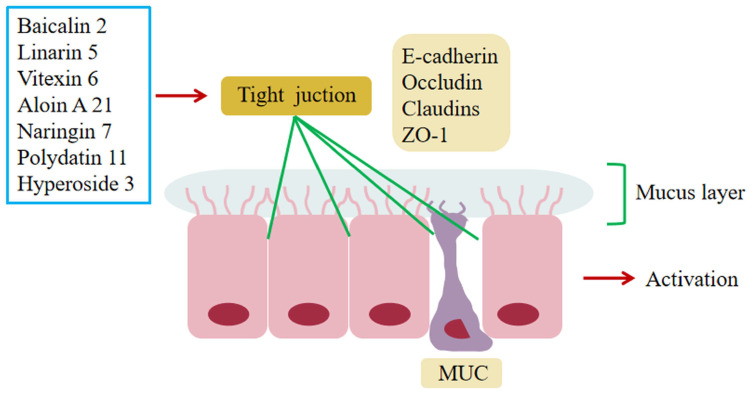
Glycosides and intestinal epithelial barrier. Polydatin **11**, baicalin **2**, hyperoside **3**, aloin A **21**, linarin **5**, vitexin **6**, and naringin **7** can improve the expression of TJ proteins and mucin proteins, thus inhibiting the increase of the permeability of the intestinal mucosa to achieve regulation of intestinal epithelial barrier function.

It was found that MUC2, MUC3A, claudin-1, occludin, and ZO-1 expression levels were significantly increased in colonic tissues of mice with colitis treated with polydatin **11**, reducing the intestinal inflammatory response [50]. In addition, baicalin **2**, hyperoside **3**, aloin A **21**, linarin **5**, vitexin **6**, and naringin **7** can improve the expression of TJ proteins and mucin proteins in intestinal mucosa and inhibit the increase of intestinal mucosal permeability, exerting anti-inflammatory effects [64,67,71,72,74,95]. The above suggests that intestinal epithelial barrier function plays an important role in the pathogenesis of UC (Figure 4).

### 6.5. Regulation of Immune Cells

CD4^+^ T cells can differentiate into Th1, Th2, Th17, and Treg cells in response to different cytokine stimuli [173]. Previous studies have shown that the pro-inflammatory effects of Th17 cells can be antagonized by Treg cells, producing the anti-inflammatory cytokines IL-10 and TGF-β [174]. Conversely, Treg can alleviate colitis by down-regulating Th1 and Th17 through IL-10 and TGF-β [175]. In addition, differentiation of both Treg and Th17 cells requires TGF-β to induce Foxp3 and RORγt. Already-differentiated Treg cells stimulated by IL-6 can inhibit Foxp3 expression and release IL-17, which in turn induces cell differentiation into Th17 cells [176]. Th17/Treg cells remain in balance under normal conditions, and once imbalanced, especially when Th17 cells are overrepresented, they can be reduced to Th17 cells. In particular, an excessive increase in Th17 cells can lead to intestinal mucosal damage and inflammatory bowel disease. Therefore, maintaining the homeostasis of Th17/Treg cells is important to prevent the development of UC.

**Figure 5:**
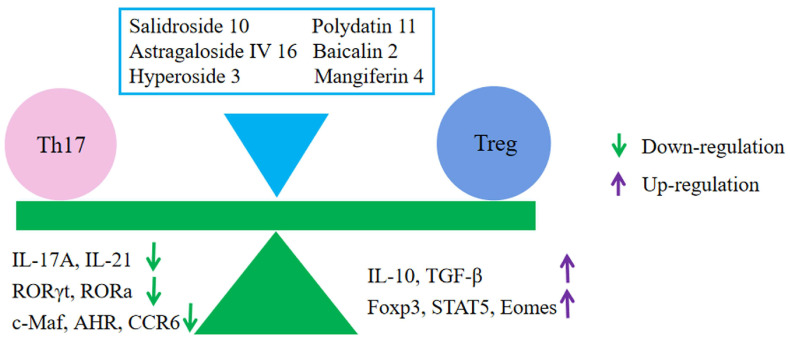
Glycosides and Th17/Treg balance. Polydatin **11**, salidroside **10**, astragaloside Ⅳ **16**, hyperoside **3**, and mangiferin **4** produced relief of UC by regulating the balance of Th17/Treg cells.

Studies have shown that polydatin **11** can reduce DSS- and TNBS-induced colitis in mice by directly binding to STAT3 and regulating Th17 cell differentiation and Th17/Treg homeostasis [81]. Meanwhile, the anti-inflammatory effects of salidroside **10**, astragaloside Ⅳ **16**, hyperoside **3**, and mangiferin **4** were also associated with the regulation of Th17/Treg cell homeostasis in experimental UC [65,69,79,90]. In addition, hyperoside **3** was also able to regulate PPARγ levels via MKRN1, thereby restoring Th17/Treg homeostasis to reduce DSS-induced colitis in mice [67] (Figure 5).

### 6.6. Regulation of UC-Related Receptors

#### 6.6.1. Inhibition of Toll-like Receptors (TLRs)

TLRs are recognition factors that initiate inflammatory responses and immune responses. When activated, they bridle MyD88 protein for signaling and promoting the expression of related inflammatory factors, exacerbating intestinal inflammation. Previous studies have demonstrated that the TLRs/MyD88/NF-κB signaling pathway plays an important role in UC [51,61,73].

**Figure 6:**
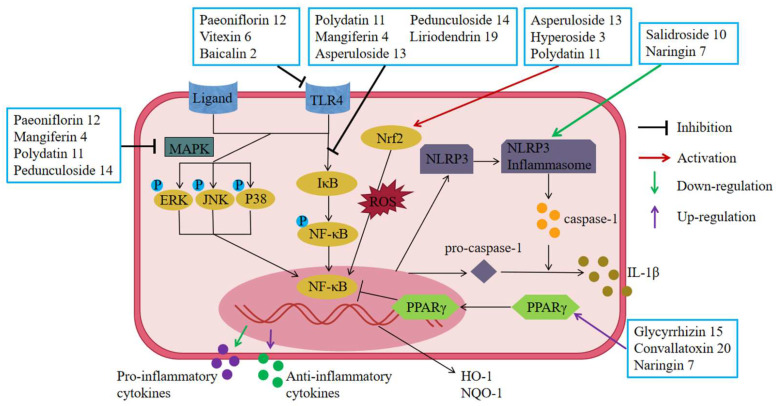
Glycosides and UC-related receptors AND transcriptional regulation. Paeoniflorin **12**, vitexin 6, and baicalin **2** produced relief of UC by reducing TLR4 expression and blocking the TLR4/NF-κB signaling pathway. Glycyrrhizin **15**, convallatoxin **20**, and naringin **7** produced relief of UC by suppressing the expression of NF-κB via activated PPARγ. Salidroside **10** and naringin **7** produced relief of UC by inhibiting the expression of inflammasome and thereby reducing the release of IL-1β. Punicalagin **8**, paeoniflorin **12**, pedunculoside **14**, liriodendrin **19**, baicalin **2**, mangiferin **4**, polydatin **11**, and vitexin **6** produced relief of UC by blocking the NF-κB signaling pathway. Mangiferin **4**, polydatin **11**, pedunculoside **14**, and paeoniflorin **12** produced relief of UC by blocking the MAPK signaling pathway. Asperuloside **13**, hyperoside **3**, and polydatin **11** produced relief of UC by blocking the Nrf2/HO-1 signaling pathway.

Paeoniflorin **12** and vitexin **6** can exert anti-inflammatory effects by reducing TLR4 expression and blocking the TLR4/NF-κB signaling pathway [51,73]. Baicalin **2** can reduce the expression of TLR2, TLR4, and TLR9 and inhibit NF-κB by blocking MyD88 signaling activation, thus inhibiting the production of inflammatory factors and exerting a protective effect [61] (Figure 6).

#### 6.6.2. Up-Regulation of Peroxisome Proliferator-Activated Receptor (PPARγ)

PPARr is a member of the nuclear receptor superfamily, most of which are ligand-dependent transcriptional activators. The anti-inflammatory activity of activated PPARγ is mediated through inhibition of NF-κB activity, leading to a reduction in the expression of inflammatory factors and exerting anti-inflammatory effects [88,94].

Glycyrrhizin **15**, convallatoxin **20**, and naringin **7** were all able to inhibit the expression of NF-κB and other inflammatory factors by increasing the expression of PPARγ, thereby reducing colonic mucosal inflammation and improving experimental colitis [74,88,94] (Figure 6).

#### 6.6.3. Inhibition of Nucleotide-Binding Oligomerization Domain (NOD)-Like Receptors (NLRs)

NOD-like receptor protein 3 (NLRP3) inflammasomes in NLRs are important regulators of intestinal homeostasis. Previous studies have shown that NLRP3 can improve experimental colitis by down-regulating IL-1β levels, and therefore, NLRP3 inflammasome is essential in the pathogenesis of UC.

Studies have shown that naringin **7** can exert anti-UC effects by down-regulating the expression of NLRP3 inflammasome [74]. Furthermore, in LPS-induced macrophages RAW264.7, salidroside **10** was similarly able to exert anti-inflammatory effects by down-regulating NLRP3 inflammasome excitation and apoptosis [79] (Figure 6).

### 6.7. Regulating Signal Transduction

#### 6.7.1. Inhibition of the NF-κB Pathway

Activation of NF-κB is a key step in the activation and proliferation of the inflammatory response in enteritis [51]. As a heterodimer complex of (p50/p65), NF-κB is regulated by IκB and IKK. Activated in response to external stimuli, IKKs phosphorylates the inhibitory IκB protein. Activated NF-KB is then transferred to the nucleus, where it binds to target DNA elements and encodes multiple inflammatory mediators.

It was shown that paeoniflorin **12** reduced NF-κB expression in DSS/TNBS-induced colonic tissue of UC rats and down-regulated inflammatory mediators in colonic mucosa [51,82,83,84]. In vitro, experiments also revealed that polydatin **11**, pedunculoside **14**, mangiferin **4**, liriodendrin **19**, and asperuloside **13** inhibited the nuclear translocation of NF-κB and thus the inflammatory response of LPS-activated macrophages RAW264.7 [50,70,85,86,93]. In addition, the mechanisms by which vitexin **6** and baicalin **2** eliminated experimental colitis were both targeted to inhibit activation of the TLR4/NF-κB pathway [61,73] (Figure 6).

#### 6.7.2. Inhibition of the MAPK Pathway

The MAPK family consists of three main members: JNK, p38MAPK, and ERK [86]. ERK is mainly activated by mitogen. p38MAPK can induce the expression of inflammatory factors such as TNF-α, ILs, and IFN-γ as well as COX-2 by mediating the activation of NF-κB [177]. JNK phosphorylates the transcription factor c-JNK and induces the production of related inflammatory factors that trigger UC. In addition, JNK activates the transcription factor STAT3 and the non-transcription factor Bcl-2, which play an important role in the development of UC [178].

In the DSS-induced UC mouse model, paeoniflorin **12** and mangiferin **4** were able to significantly inhibit the increased phosphorylation levels of ERK1/2, JNK, and p38, exerting anti-inflammatory effects [70,82,83]. In in vitro experiments, polydatin **11** and pedunculoside **14** also significantly inhibited the phosphorylation of ERK1/2, JNK1/2, and p38, thereby reducing the production of IL-1β, IL-6, TNF- α, COX-2, and iNOS and suppressing the inflammatory response [50,86] (Figure 6).

#### 6.7.3. Inhibition of the Nrf2/HO-1 Pathway

The transcription factor Nrf2 is the most important transcription factor against oxidative stress and maintains mucosal homeostasis by inhibiting the production of excess ROS. When activated, Nrf2 is phosphorylated, leading to increased expression of antioxidant genes such as SOD, NQO1, CAT, GSH-Px, and HO-1 [50,85].

Asperuloside **13** can reduce DSS-induced oxidative stress and inflammation in mouse colon tissue by activating the Nrf2/HO-1 signaling pathway; increasing the expression of Nrf2, HO-1, and NQO-1 proteins; and down-regulating p65 levels [85]. Hyperoside **3** was also able to ameliorate the inflammatory response and apoptosis in experimental colitis by activating the Nrf2 signaling pathway [66]. In vitro, experiments have also shown that polydatin **11** can exert anti-inflammatory effects by inhibiting MAPK and NF-κB inflammatory signaling pathways and activating AKT/Nrf2/HO-1/NQO1 signaling pathway [50] (Figure 6).

#### 6.7.4. Inhibition of Other Related Pathways

The pathogenesis of UC also involves the JAK-STAT, PI3K/AKT, Notch, and Shh pathways. Through in vivo and vitro experiments of UC, polydatin **11** was able to specifically block the JAK/STAT3 signaling pathway and inhibit the differentiation of Th17 cells to improve intestinal inflammation [81]. In TNBS-induced colitis, baicalin **2** was able to play a protective role in colitis by inhibiting the PI3K/AKT pathway [62]. Astragaloside Ⅳ **16** and aloin A **21** can effectively inhibit DSS-induced inflammatory damage in colon tissue by inhibiting the Notch signaling pathway and reconstructing the colonic mucosa [90,95]. Oxidative stress and epithelial cell apoptosis in the gastrointestinal tract are associated with the Shh pathway, and polydatin **11** may inhibit experimental colitis by modulating the Shh signaling pathway [47].

## 7. Conclusions and Outlook

A large number of review papers discuss the possible application of various extracts from natural products as well as pure biologically active substances in medicine [179,180,181,182].

Pure bioactive substances include synthetic drugs and pure natural substances. The decisive factor for the dominance of pure bioactive substances in modern medicine is the ease of drug administration and clinical pharmacological evaluation. Synthetic drugs have been widely used in clinical practice, but there are many side effects as well as resistance and drug-induced diseases; in addition, the development cycle of synthetic drugs is long, the investment is large, and the enterprises often cannot bear it.

With the progress of science, people’s awareness of self-care is enhanced, the understanding of natural medicine is deepened, and the desire to return to nature is rising, so the demand for natural medicine is increasing [183].

Natural products are the largest pool of biologically active substances on Earth [179]. The bioactive compounds derived from natural products, especially medicinal plants, have emerged as new treatments for a variety of diseases. Natural products with biodiversity and chemical diversity, especially higher plants, will always be the most reliable source of leads for the development of new drugs [13]. In addition, the success rate of the development of new drugs by synthetic substances is extremely low, while the success rate of the development of new drugs by natural products from plants is much higher, the development time is greatly shortened, and the financial and human investment is also reduced accordingly.

Despite the great potential of natural products in the treatment of diseases, there are other issues that need to be addressed, such as the extraction, isolation, and standardization of derived compounds as well as their effective treatment modalities. In addition, some natural ingredients have low activity, a narrow antibacterial spectrum, strong drug resistance, poor stability, or serious side effects, and the corresponding technology should be used for structural modification to overcome their defects. Therefore, much research is needed before any natural product can be used as a treatment [13].

The extraction of active ingredients from natural products is an important source of new drug discovery. Understanding the mechanism of action of drugs helps to obtain the best clinical treatment drugs. This manuscript reviews 21 glycosides with relatively clear anti-inflammatory effects and mechanisms of action to provide new insights and ideas for the discovery of new drugs for the treatment of UC.

**Figure 7:**
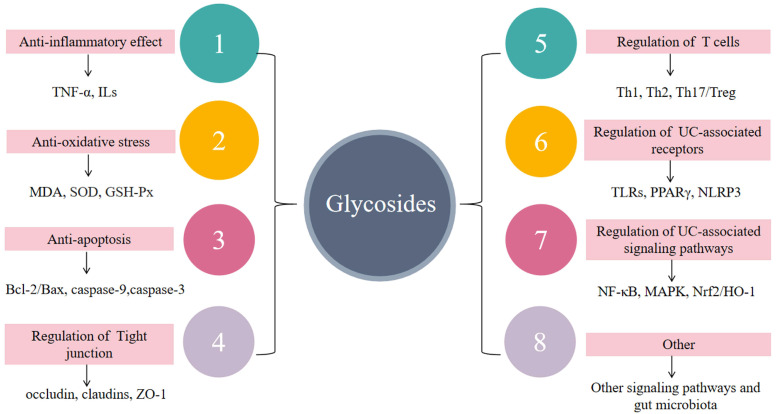
The mechanism of glycosides in the treatment of UC. Glycosides play a role in the treatment of UC through anti-inflammatory and antioxidant stress mechanisms, regulation of impaired intestinal epithelial barrier function, regulation of immune cells, and regulation of UC-related receptors and signal transduction.

Glycosides have a wide range of pharmacological effects, including antioxidant, analgesic, antipyretic, anti-hypertensive, anti-tumor, and neuroprotective effects in addition to anti-inflammatory effects [71,113,114,115,116,117]. In this review, we found that glycosides can improve UC symptoms and play a role in the prevention and treatment of UC by inhibiting inflammatory response, reducing oxidative stress and anti-apoptosis, inhibiting abnormal immune response, and regulating signal pathway transduction (Figure 7). The glycosides in this review mainly include flavonoids, phenols, terpenes, etc., which can exert anti-inflammatory effects by inhibiting the expression of inflammatory-related factors and reducing oxidative stress. The main mechanisms include NF-κB, MAPK inflammatory signaling pathways, and the Nrf2/HO-1 signaling pathway. Astragaloside Ⅳ **16** and aloin A **21** can inhibit the colonic inflammatory response by modulating the Notch signaling pathway. Polydatin **11** was also able to reduce the inflammatory response by acting on JAK/STAT3 and Shh signaling pathways. Baicalin **2** can play a protective role in colitis by inhibiting the PI3K/AKT pathway. In addition, the intestinal flora also plays an important role in the pathogenesis of UC, but there are few studies on glycosides in this regard, which need further study. Meanwhile, glycosides are also worthy of further study in the prevention and treatment of UC-related carcinogenesis.

UC is a complex disease, and although we have identified a strong relationship between glycosides and UC, there are still many issues to be resolved. Firstly, oral bioavailability is poor, and glycosides must be hydrolyzed by intestinal enzymes or microflora before they can be absorbed. Secondly, glycosides are unstable and readily hydrolyzed by acids, bases, and enzymes. It has been found that only 10–15% of the glycosides are absorbed in the small intestine, with the remaining glycosides being metabolized by microorganisms in the large intestine to form small molecules, so it is doubtful whether it is the broken-down glycosides and their metabolites or the original glycoside molecules that work in the body [184,185,186]. Thirdly, the side effects of glycosides and the pharmacological control of UC complications are not fully understood.

In summary, these glycosides are valuable candidates for the prevention and treatment of UC even taking into account the problems mentioned above. Therefore, it is urgent to study the pharmacokinetic characteristics of natural-product-isolated glycosides and establish their dose–pharmacology–toxicology relationship. Further clinical studies are essential to prove the effective role of glycosides in the prevention and treatment of UC. Therefore, future research must explore the targets and molecular mechanisms of glycosides, combine laboratory anti-UC studies with clinical practice, test the reliability of glycosides against UC, and promote their use in the practical prevention and treatment of UC.

## Figures and Tables

**Table 1:** Drugs commonly used in the treatment of UC.

Types	Drugs	Subjects Treated	Side Effects	References
Aminosalicylates	Salazopyridine 5-Aminosalicylic acid Olsalazine Mesalazine	First-line drugs used to treat mild and moderate UC.	Long-term use can lead to drug resistance and may cause adverse effects such as damage to the blood, liver, kidney, and digestive tract and folic acid deficiency.	[22,23]
Glucocorticoids	Prednisone Budesonide Beclomethasone Fluticasone propionate	For acute and severe UC and mild patients who are intolerant or refractory to aminosalicylates.	Causes metabolic disorders, osteoporosis, etc. Long-term use can lead to drug dependence and irreversible complications.	[24,25]
Immunosuppressants	Azathioprine Methotrexate Tacrolimus	For the palliative treatment of hormone-dependent UC patients and severe cases that do not respond to steroids.	Hepatotoxic and nephrotoxic, may increase the risk of infection, and generally used clinically only as an adjunct.	[26,27]
Microbiological agents	*Lactobacillus rhamnosus* GG	For improving the symptoms of mild to moderate UC relapses.	Risk of bacterial translocation and subsequent bacteremia.	[28,29]
Biological agents	Infliximab for Injection Tofacitinib	For patients with acute severe UC; for patients with severe UC where immunosuppressive drugs are ineffective or active UC with severe extraintestinal manifestations.	It is very effective for severe patients, but its use is limited due to its high price and side effects such as leukopenia, neutropenia, and allergy	[30,31,32]

**Table 2:** Classifications and Chemical Characteristics of Glycosides.

No.	Glycosides	Classifications	CAS	Molecular Formulas
**1**	Quercitrin	Flavonoids	522-12-3	C_21_H_20_O_11_
**2**	Baicalin	Flavonoids	21967-41-9	C_21_H_18_O_11_
**3**	Hyperoside	Flavonoids	482-36-0	C_21_H_20_O_12_
**4**	Mangiferin	Flavonoids	4773-96-0	C_19_H_18_O_11_
**5**	Linarin	Flavonoids	480-36-4	C_28_H_32_O_14_
**6**	Vitexin	Flavonoids	3681-93-4	C_21_H_20_O_10_
**7**	Naringin	Flavonoids	10236-47-2	C_27_H_32_O_14_
**8**	Punicalagin	Phenolics	65995-63-3	C_48_H_28_O_30_
**9**	Curculigoside	Phenolics	85643-19-2	C_22_H_26_O_11_
**10**	Salidroside	Phenolics	10338-51-9	C_14_H_20_O_7_
**11**	Polydatin	Phenolics	65914-17-2	C_20_H_22_O_8_
**12**	Paeoniflorin	Terpenoids	23180-57-6	C_23_H_28_O_11_
**13**	Asperuloside	Terpenoids	14259-45-1	C_18_H_22_O_11_
**14**	Pedunculoside	Terpenoids	42719-32-4	C_36_H_58_O_10_
**15**	Glycyrrhizin	Terpenoids	1405-86-3	C_42_H_62_O_16_
**16**	Astragaloside Ⅳ	Terpenoids	84687-43-4	C_41_H_68_O_14_
**17**	Gentiopicroside	Terpenoids	20831-76-9	C_16_H_20_O_9_
**18**	Ginsenoside Rg1	Terpenoids	22427-39-0	C_42_H_72_O_14_
**19**	Liriodendrin	Lignans	573-44-4	C_34_H_46_O_18_
**20**	Convallatoxin	Steroids	508-75-8	C_29_H_42_O_10_
**21**	Aloin A	Anthraquinones	1415-73-2	C_21_H_22_O_9_

**Table 3:** A list of glycosides with inhibitory effects on UC.

Glycosides	Animal	Model	Dose	Effects/Mechanisms of Action					References
				Behavioral Evaluation	Colon Length	Histopathological Evaluation	Biochemical/Molecular Parameters/mRNA	Related Molecular Mechanisms	
Quercitrin **1**	Male Wistar–Albino rats	DDS- induced colitis	1 and 5 mg/kg i.g. 10 days			↓ Colon tissue damage	↓ MPO and TNF-α		[59]
	Female Wistar rats	TNBS- induced colitis	1 and 5 mg/kg p.o. once			↓ Colon tissue damage	↓ iNOS, COX-2, NOX1, TNF-α, and IL1β		[60]
Baicalin **2**	Female C57BL/6 (B6) mice/ RAW264.7 macrophages	DDS- induced colitis/LPS-induced inflammatory macrophage model	100 mg/kg i.g. twice daily for 7 days	↓ DAI		↓ Histological score	↓ TNF-α, IL-6, and IL-13 ↓ MyD88, NF-κB p65 ↓ TLR2, TLR4, and TLR9 ↑ IL-10	↓ TLR4/NF-κB-p65 pathway	[61]
	Sprague–Dawley rats	TNBS- induced colitis	100 mg/kg i.g. 14 days				↓ p-PI3K/PI3K, p-AKT/AKT, TNF-α,IL-6, and IL-1β ↑ IL-10 and ZO-1	↓ PI3K/AKT pathway	[62]
	Male Sprague–Dawley rats/ RAW264.7 macrophages	TNBS- induced colitis/LPS-induced inflammatory macrophage model	30, 60 and 120 mg/kg i.g. 14 days	↓ DAI	↓ Colonic shortening	↓ Histological score	↓ MDA ↓ TGF-β1, Bax, and ROS ↓ Caspase-3, cleaved caspase-3, caspase-9, cleaved caspase-9, Fas, and FasL ↑ CAT, GSH-Px, and SOD ↑ Bcl-2	↓ Oxidant stress and apoptosis	[63]
	Male C57BL/6J mice	DDS- induced colitis	20, 50 and 100 mg/kg i.g. 7 days	↓ DAI		↓ Histological score	↓ TNF-α, IL-6, and IL-1β ↓ caspase-1 and claudin-2 ↑ IL-10 ↑ ZO-1, NLRP6, MUC2, ASC, and IL-18 ↑ E-cadherin, claudin-4, and claudin-5	↑ NLRP6/IL-18 pathway	[64]
	SD rats	TNBS- induced colitis	10 mL/kg i.g. twice daily for 7 days	↓ Weight loss ↓ DAI	↓ Colonic shortening	↓ Histological score	↓ MPO ↓ TNF-α, IL-1β, IL-6, IL-17, and IL-12 ↓ RORγt and Th17/Treg ↑ TGF-β, IL-10, and Foxp3		[65]
Hyperoside **3**	Male C57BL/6 mice	DDS- induced colitis	80 and 120 mg/kg i.g. 14 days	↓ DAI	↓ Colonic shortening	↓ Histological score	↓ TNF-α, IL-6, COX-2, and NF-κB p65 ↓ MDA ↓ Caspase-3 and Bax ↑ IL-10 ↑ Bcl2 ↑ Nrf2, HO-1, and SOD	↑ Nrf2 pathway	[66]
	Male C57BL/6 mice	DDS- induced colitis	3, 10 and 30 mg/kg p.o. 7 days/3, 10, and 30 μM	↓ Weight loss ↓ DAI	↓ Colonic shortening	↓ Histological score	↓ TNF-α, IL-1β, IL-6, IL-17, and IL-22 ↓ MKRN1, RORγt, and Th17/Treg ↑ ZO-1, claudin-5, and MUC2 ↑ Foxp3, IL-10, and TGF-β		[67]
Mangiferin **4**	Male Wistar rats	TNBS- induced colitis	10, 30, and 200 mg/kg i.g. 16 days	↓ Weight loss		↓ Structural distortion of crypts, desquamated areas or loss of epithelium, and goblet cell depletion	↓ TNF-a, IL-17, MDA, and SOD		[68]
	Male C57BL/6 mice	TNBS- induced colitis	10 and 20 mg/kg p.o. 3 days	↓ Weight loss	↓ Colonic shortening		↓ MPO ↓ TNF-α, IL-17, NF-κB, iNOS, and COX-2 ↓ Th17, IL-17, RORγt, and STAT3 ↑ Treg ↑ Foxp3, IL-10, and STAT5		[69]
	Female C57BL/6 mice/ RAW264.7 macrophages	DDS- induced colitis/LPS-induced inflammatory macrophage model	50 mg/kg p.o. 13 days	↓ Weight loss	↓ Colonic shortening	↓ Histological score	↓ MPO ↓ TNF-α, IκBα, p-IκBα, p-p65NF-κB, iNOS, ICAM-1, IL-1β, IL-6, p-ERK1/2, ERK1/2, p-JNK, JNK, p-p38MAPK, and p38MAPK	↓ NF-κB and MAPK pathways	[70]
Linarin **5**	Male C57BL/6J mice	DDS- induced colitis	25 and 50 mg/kg i.g. 14 days	↓ Weight loss ↓ DAI	↓ Colonic shortening	↓ Histological score	↓ MPO ↓ IL-6, TNF-α, IFN-γ, and IL-1β ↑ IL-10 ↑ ZO-1, Occludin, and Claudin-1		[71]
Vitexin **6**	Male BALB/c mice	DDS- induced colitis	20 and 80 mg/kg i.g. 7 days	↓ Weight loss ↓ DAI	↓ Colonic shortening	↓ Histological score	↓ IL-1β, IL-6, TNF-α, p-p65/p65, pIκB/IκB, and p-STAT1/STAT1 ↑ IL-10 ↑ MUC2, ZO-1, and Occludin		[72]
	Male BALB/C mice	DDS- induced colitis	40 and 80 mg/kg p.o. 7 days			↓ Histological scores of liver	↓ TNF-α, IL-6, and IL-1β ↓ ALT, TC, AST, and TG ↓ TLR4, NF-κB p65, p-p65, IκBα, and p-IκBα	↓ TLR4/NF-κB pathway	[73]
Naringin **7**	Male C57BL/6 mice	DDS- induced colitis	25, 50, and 100 mg/kg p.o. 7 days	↓ DAI	↓ Colonic shortening	↓ Histological score	↓ TNF-α, IL-1β, and IL-6 ↓ p-p65NF-κB, p-IκBα, p-p38MAPK, p-ERK, and p-JNK ↓ NLRP3, ASC, and Caspase-1 ↑ PPARγ and ZO-1		[74]
	Male Wistar rats	TNBS- induced colitis	20, 40, and 80 mg/kg p.o. 14 days	↓ Weight loss ↓ Rectal bleeding ↓ The ratio of colon weight/colon length ↓ Diarrhea score		↓ Histological score	↓ MDA and MPO ↓ TNF-a and IL-12 ↓ SGPT, SGOT, and ALP ↑ SOD, GSH-Px, and CAT		[75]
Punicalagin **8**	Male SD rats	DNBS- induced colitis	4 mg/kg p.o. 18 days	↓ DAI		↓ CMDI	↓ MPO, MDA, and NO ↓ TNF-α, IL-1β, IL-18, and NF-κB		[76]
Curculigoside **9**	Male C57BL/6J mice	DDS- induced colitis	50 and 100 mg/kg p.o. 7 days	↓ Weight loss ↓ DAI	↓ Colonic shortening	↓ Histological score	↓ Iron overload ↓ ROS and MDA ↑ GSH, GPX4, and SOD		[77]
Salidroside **10**	Male C57BL/6 mice	DDS- induced colitis	20 and 40 mg/kg i.g. 7 days	↓ Weight loss ↓ DAI	↓ Colonic shortening	↓ Colon tissue damage	↓ Bax, caspase-3, and cleaved-caspase-3 ↑ SOD, GSH-Px, and CAT ↑ Bcl-2 ↑ SIRT1, FoxO1, FoxO3a, and FoxO4	↑ SIRT1/FoxOs pathway	[78]
	Male C57BL/6 mice/ RAW264.7 macrophages	DDS- induced colitis/LPS-induced inflammatory macrophage model	7.5, 10, and 15 mg/kg i.g. 7 days/10, 20, 40, and 80 μM	↓ Weight loss ↓ DAI	↓ Colonic shortening	↓ Colonic mucosal erosion, crypt loss, and extensive lymphocyte infiltration	↓ MPO ↓ IL-1β, IL-6, IFN-γ, and IL-17A ↓ NLRP3, caspase-1, TREM1, DAP12, and GSDMD p30 ↓ Th17 ↑ Treg	↓ TREM1 signal cascade ↓ Th17/Treg imbalance	[79]
Polydatin **11**	Male C57BL/6 mice	DDS- induced colitis	15, 30, and 45 mg/kg i.p. 7 days	↓ Weight loss ↓ DAI	↓ Colonic shortening	↓ Histological score	↓ MDA ↓ Caspase 3, cleaved caspase 3, and Bax ↑ SOD and GSH-Px ↑ Bcl-2 ↑ Shh, Ptc, Smo, and Gli1	↓ Oxidative stress and apoptosis ↑ Shh pathway	[47]
	Male Wistar rats	Acetic-acid- induced colitis	45 mg/kg p.o. 10 days	↓ DAI ↓ Adhesion score		↓ Histological score	↓ MPO, IL-1β, TNF-α, and IL-6 ↑ SOD and GSH-Px ↓ Caspase 3	↓ Oxidative stress and apoptosis partially	[80]
	C57BL/6 mice/ RAW264.7 macrophages	DDS- induced colitis/LPS-induced inflammatory macrophage model	p.o. 11 days /100, 200, 300, and 400 μM	↓ Weight loss ↓ DAI	↓ Colonic shortening	↓ Histological score	↓ TNF-α, IL-6, IL-4, iNOS, and COX-2 ↓ ERK1/2, JNK1/2, and p38 ↑ IL-10 ↑ Claudin-1, Occludin, ZO-1, MUC2, and MUC3A ↑ AKT, Nrf2, HO-1, and NQO-1	↓ Oxidative stress ↓ NF-κB and MAPK pathways ↑ AKT/NF-κB/NQO-2/HO-1pathway	[50]
	Male C57BL/6J mice	DDS- induced colitis/ TNBS- induced colitis	30 and 60 mg/kg i.g. 10 days/5 days	↓ Weight loss ↓ DAI	↓ Colonic shortening	↓ Histological score	↓ TNF-α and IL-17A ↓ Th17/ Treg cells ↑ Occludin	↓ JAK/STAT pathway	[81]
Paeoniflorin **12**	Female C57BL/6 mice	DDS- induced colitis	50 mg/kg p.o. 10 days				↓ MPO, TNF-α, and IL-6 ↓ NF-κB, ERK1/2, JNK, and p38 MAPKs	↓ MAPK/NF-κB pathway	[82]
	Female C57BL/6 mice	AOM/DSS-induced CAC model	3 g/kg p.o. 28 days				↓ TNF-α, IL-1β, IL-6, IL-13, NF-κB, TLR4, and EGFL7	↓ TLR4/NF-κB pathway	[51]
	Male Balb/c mice	TNBS-induced colitis	15, 30, and 45 mg/kg p.o. 7 days	↓ Weight loss	↓ Colonic shortening	↓ Colonic damage of macroscopic scores	↓ MPO, IL-2, IL-6, IL-10, IL-12, IL-1β, TNF-α, and IFN-γ ↓ Bax, cytochrome c, caspase 3, and caspase 9 ↓ p-JNK/JNK ↑ p-P38/P38, p-ERK/ERK, p-NF-κB/NF-κB, and p-IκBα/IκBα ↑ Bcl-2	↓ MAPK/NF-κB pathway ↓ Apoptosis	[83]
	Male C57BL/6 mice	DDS- induced colitis	20 mg/kg p.o. 7 days	↓ Weight loss	↓ Colonic shortening ↓ Increased spleen weight	↓ Eosinophil infiltration	↓ Eosinophil infiltration ↓ Inflammatory cytokines ↑ Treg, p-STAT3, and CCR3 ↑ Eotaxin	↓ NF-κB pathway	[84]
Asperuloside **13**	Male KM mice / RAW264.7 macrophages	DDS- induced colitis/ LPS-induced inflammatory macrophage model	0.125 0.5 mg/kg p.o. 38 days	↓ Weight loss ↓ DAI	↓ Colonic shortening and increased colon thickness	↓ Inflammatory cell infiltration, epithelial cell destruction, mucosal thickening, and lower microscopic score	↓ MPO and MDA ↓ TNF-α, IL-6, and NF-κB ↑ SOD and GSH-Px ↑ Nrf2, HO-1, and NQO-1	↓ Oxidative stress and NF-κB activation ↑ Nrf2/HO-1 pathway	[85]
Pedunculoside **14**	Male C57BL/6 mice/ RAW264.7 macrophages	DDS- induced colitis/ LPS-induced inflammatory macrophage model	5, 15, and 30 mg/kg p.o. 7 days		↓ Colonic shortening	↓ Loss of goblet cells and crypts, increased inflammatory tissue infiltration, and severe destruction of colon structure	↓ MPO ↓ AKT, ERK1/2, JNK1/2, p65, and p38 ↓ IL-1β, IL-6, TNF- α, COX-2, iNOS, and NF-κB	↓ MAPK and AKT/NF-κB pathways	[86]
Glycyrrhizin **15**	Female SD rats	Acetic-acid- induced colitis	40 mg/kg i.p. 7 days	↓ DAI		↓ Morphologic injury and histological changes	↓ MPO ↓ NF-κB, TNF-α, and ICAM-1		[87]
	Albino Wistar rats	Acetic-acid- induced colitis	100 mg/kg p.o. 8 days			↓ Colonic tissue injury	↓ MPO ↓ TNF-α ↑ SOD, GSH-Px, and CAT ↑ PPARγ		[88]
	Male BABL/c mice	TNBS- induced colitis	50 mg/kg i.p. once every 2 days for 5 days	↓ Weight loss ↓ DAI	↓ Colonic shortening	↓ Histological score	↓ HMGB1, IFN-γ, IL-6, and TNF-α ↓ Th17, Th1, CDs		[89]
Astragaloside Ⅳ **16**	Male C57BL/6 mice	DDS- induced colitis	50 and 100 mg/kg i.g. 7 days	↓ Weight loss ↓ DAI	↓ Colonic shortening ↓ Colon weight	↓ Histological score	↓ AHR, c-Maf, RORa, and RORrt ↓ CCR6 ↓ IL-17A, IL-21 ↓ Eomes, Foxp3, and STAT5 ↓ MDA ↓ DLL3, Jagged1, Jagged2, Notch2, Notch3, Hes1, and Hes2 ↑ IL-10 and TGF-β1 ↑ CAT, SOD, and GSH-Px	↓ Oxidative stress ↓ Th17/Treg ↓ Notch pathway	[90]
Gentiopicroside **17**	Male ICR mice	DDS- induced colitis	50, 100, and 200 mg/kg i.g. 7 days	↓ DAI	↓ Colonic shortening	↓ Histological score	↓ MPO ↓ TNF-α, IL-1β, IL-6, iNOS, and COX-2		[91]
Ginsenoside Rg1 **18**	Male C57BL/6 mice	DDS- induced colitis	200 mg/kg p.o. 10 days	↓ Weight loss	↓ Colonic shortening	↓ Histological score	↓ IL-2 and TNF-α		[92]
Liriodendrin **19**	Male BALB/c mice/ RAW264.7 macrophages	DDS- induced colitis/LPS-induced inflammatory macrophage model	100 mg/kg i.g. 10 days	↓ DAI	↓ Colonic shortening	↓ Histological damage	↓ MPO and MDA ↓ TNF-α IL-1β and IL-6 ↑ SOD and GSH-Px ↑ ERβ	↓ Akt and NF-κB pathways	[93]
Convallatoxin **20**	Female C57BL/6 mice/ RAW264.7 macrophages	DDS- induced colitis/LPS-induced inflammatory macrophage model	50 and 150 μg/kg	↓ Weight loss ↓ DAI	↓ Colonic shortening		↓ TNF-α, IL-1β, and IL-6 ↓ NF-κB-p65 and IκBα ↓ COX-2 and iNOS ↑ PPARγ		[94]
Aloin A **21**	Male C57BL/6J mice/ RAW264.7 macrophages	DDS- induced colitis/LPS-induced inflammatory macrophage model	25 and 50mg/kg i.g. 7days	↓ Weight loss ↓ DAI	↓ Colonic shortening	↓ Histological score	↓ MPO ↓ IL-1β, TNF-α, and IL-6 ↓ Cleaved caspase-3 ↓ Notch1 and Hes1 ↑ IL-10 ↑ Ki-67 ↑ MUC2, ATOH1, ZO-1, and Occludin	↓ Notch pathway	[95]

SD, Sprague–Dawley; ICR, Institute of Cancer Research; DSS, dextran sulfate sodium; DNBS, 2,4-Dinitro benzene sulfonic acid; TNBS, trinitrobenzene sulfonic acid; CMDI, colon mucosa damage index; DAI, disease activity index; AOM, azoxymethane; CAC, colitis-associated cancer; i.p., intraperitoneal injection; i.t., intrathecal injection; p.o., oral; i.g., intragastrically. ↑, enhanced/increased/up-regulated; ↓, attenuated/down-regulated/decreased/suppressed/inhibited/prevented/improved.

## Data Availability

The data that support the findings of this study are available from the corresponding author upon reasonable request.

## Abbreviations

IBD	Inflammatory bowel disease
TNF-α	Tumor necrosis factor-alpha
IL	Interleukin
MAPK	Mitogen-activated protein kinase
NF-κB	Nuclear factor kappa-B
STAT	Signal transducer and activator of transcription
Treg	Regulatory T cells
IFN-γ	Interferon-γ
ERK	Extracellular signal-regulated kinase
JNK	C-Jun N-terminal kinase
TLR4	Toll-like receptor4
EGFL7	Epidermal growth factor-like domain 7
MPO	Myeloperoxidase
MDA	Malondialdehyde
GSH-Px	Glutathione peroxidase
GPX4	Anti-oxidant enzyme glutathione peroxidase 4
HO-1	Heme oxygenase-1
NQO1	Quinone oxidoreductase 1
Nrf2	Nuclear factor (erythroid-derived 2)-like 2
ROS	Reactive oxygen species
SOD	Superoxide dismutase
COX-2	Cyclooxygenase-2
INOs	Inducible nitric oxide synthase
ERβ	Estrogen receptor-β
ICAM-1	Intercellular cell adhesion molecule-1
PPARγ	Peroxisome proliferator-activated receptor γ
HMGB1	High-mobility group box 1
DCs	Dendritic cells
IFN-γ	Interferon γ
NOX1	Nicotinamide adenine dinucleotide phosphate oxidase 1
Shh	Sonic hedgehog
Ptch1	Patched1
Smo	Smoothened
Gli1	Glioma-associated oncogene homolog 1
ERK	Extracellular signal-regulated kinase
JNK	C-Jun N-terminal kinase
LPS	Lipopolysaccharide
MUC	Mucin
Th	T-helper
Tregs	Regulatory T cells
c-Maf	c-Musculoaponeurotic fibrosarcoma
AhR	Aryl hydrocarbon receptor
EOMES	Recombinant eomesodermin
FOXP3	Forkhead box P3
CCR6	Chemokine receptor 6
TGF-β1	Transforming growth factor-β1
CAT	Catalase
DLL3	Delta-like protein 3
PI3K	Phosphoinositide 3-kinase
AKT	Protein kinase B
PTEN	Phosphatase and tensin homologue deleted on chromosome ten
ROS	Reactive oxygen species
RORγt	Retinoic acid-related orphan receptor gamma t
ZO	Zonula occludens
IEC	Intestinal epithelial cell
ASC	Amino acid transporter 1
NLRP6	Nod-like receptor pyrin domain-containing protein 6
MKRN1	Makorin ring finger protein 1
OCLN	Occludin
ATOH1	Recombinant human atonal homolog 1
ICAM-1	Intercellular adhesion molecule-1
IκBα	Inhibitory κB-α
ALT	Alanine aminotransferase
AST	Aspartate aminotransferase
TC	Total cholesterol
TG	Triglyceride
ASC	Apoptosis-associated particulate protein
SGOT	Serum glutamic-oxaloacetic transaminase
SGPT	Serum glutamic pyruvic transaminase
ALP	Alkaline phosphatase
TJ	Tight junction
Cyt-C	Cytochrome c
